# Hydrogel-integrated osteogenic microtissues promote repair of infected intervertebral defects through sequential immunomodulation

**DOI:** 10.1016/j.mtbio.2026.103657

**Published:** 2026-09-07

**Authors:** Shaokang Huang, Linli Li, Yichang Xu, Jianming Huang, Yi Zhang, Shuying Chen, Yu Chen, Yushu Bai, Jie Shao

**Affiliations:** a Department of Orthopedics, Changhai Hospital, Naval Medical University, Shanghai, 200433, China; b Department of Orthopedics, Huashan Hospital, Fudan University, Shanghai, 200040, China; c Department of Orthopedics, Shanghai Fifth People's Hospital, Fudan University, Shanghai, 200240, China; d Department of Laboratory Medicine, Huashan Hospital, Fudan University, Shanghai, 200040, China

**Keywords:** Intervertebral infection, Osteogenic bone microtissues, Glycyrrhizic acid hydrogel, Zinc ions, Black phosphorus, Sequential immunomodulation, Osteogenesis

## Abstract

Intervertebral infection remains difficult to treat because the adult intervertebral disc is avascular, hypoxic, and poorly perfused, limiting drug penetration, immune-cell trafficking, and metabolic waste clearance. Solving this problem requires localized control of bacteria while reconstructing a microenvironment capable of supporting vascularized bone regeneration. We developed an injectable system that integrates active bone microtissues into a hydrogel with antibacterial and immune-regulatory functions, combining local microenvironment modulation with the delivery of active osteogenic units. Within the "soil-seed-nutrient" framework, the GA/SilMA matrix serves as the immunomodulatory "soil," the osteogenic bone microtissue at day 7 acts as the active "seed," and Zn^2+^ and BP function as "nutrient" providing antibacterial and regenerative effects. This composite hydrogel reduces bacterial growth and prevents biofilm formation, while triggering a time-dependent macrophage response characterized by an initial antibacterial inflammatory phase followed by a later pro-repair state. Zn^2+^ and black phosphorus additionally supported endothelial activity and osteogenic differentiation under inflammatory conditions. In a rat Co2/Co3 intervertebral defect model, after acute *Staphylococcus aureus* contamination and early implantation of the material, GA-Zn^2+^/BP/BO@SilMA demonstrated enhanced bone formation at 12 weeks and achieved more significant structural bridging as evidenced by X-ray and histological evaluations. Transcriptome analysis revealed putative associations with immune remodeling, extracellular matrix interactions, metabolic adaptation, and osteogenic signaling pathways. Collectively, this strategy integrates multiple synergistic functions, including antibacterial activity, macrophage modulation, angiogenesis promotion, and osteogenesis, offering potential as a local adjuvant for early intervention and regenerative repair of bacterial-contaminated intervertebral defects.

## Highlights

• An injectable GA–Zn^2+^/BP/BO@SilMA hydrogel–microtissue composite was fabricated.
• A soil–seed–nutrient therapeutic concept was proposed for intervertebral infection.
• GA-Zn^2+^/BP@SilMA suppresses bacterial growth and biofilm formation.
• The hydrogel induces early antibacterial and later pro-reparative immune states.
• GA-Zn2+/BP/BO@SilMA enhances bone bridging in infected intervertebral defects.

## 1 Introduction

**Scheme 1:**
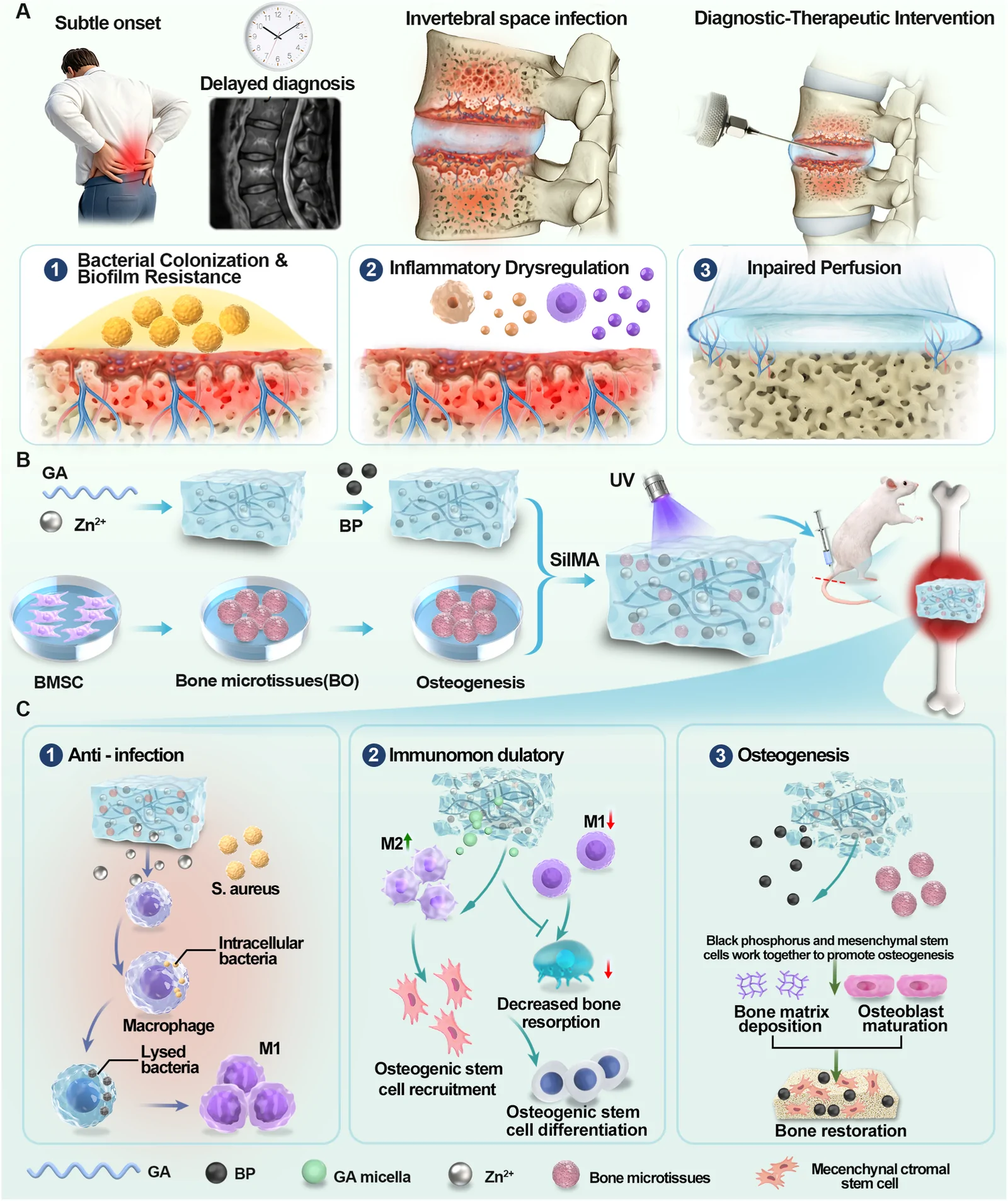
Schematic illustration of hydrogel-integrated bone microtissues for infected intervertebral defect repair. (A) Pathological challenges of intervertebral infection. (B) Fabrication of the hydrogel-integrated bone microtissues system. (C) Coordinated antibacterial, immunomodulatory, and osteogenic effects of the composite system.

Intervertebral infection, commonly presenting as pyogenic spondylodiscitis, involves the intervertebral disc, adjacent endplates, and vertebral bodies and may extend into the paravertebral soft tissues or epidural space [1,2]. Its insidious onset and nonspecific early manifestations frequently delay diagnosis. Although prolonged antimicrobial therapy and surgery are effective in many patients, recurrence, treatment failure, persistent pain, deformity, and functional impairment remain clinically important, particularly in older or medically vulnerable populations [[3], [4], [5]]. Successful treatment is constrained by the local anatomy and physiology of the intervertebral compartment. The adult intervertebral disc is avascular and hypoxic and depends largely on diffusion across the cartilaginous endplates for nutrient transport and waste removal [6,7].These diffusion barriers can limit sustained antimicrobial exposure at the infection site [8]. In parallel, *Staphylococcus aureus* and other pathogens can form biofilms that reduce antibiotic susceptibility and evade host immune clearance, while infection-driven inflammation accelerates endplate destruction, tissue necrosis, and impaired bone formation [[9], [10], [11]]. Thus, infection control alone may not restore a microenvironment capable of supporting vascularized bone repair [12] (see Scheme 1)

The transition from bacterial control to tissue regeneration is temporally coordinated. A transient pro-inflammatory macrophage response can support early bacterial clearance, whereas persistent inflammatory activation maintains oxidative stress and suppresses endothelial and osteogenic function [8,[13], [14], [15], [16], [17]]. During later repair, a shift toward pro-resolving and reparative macrophage states is required to restore immune homeostasis [18,19]. Revascularization is similarly essential because newly formed vessels provide oxygen, nutrients, progenitor-cell recruitment, and angiocrine signals that couple angiogenesis with osteogenesis [[20], [21], [22]].

To address these sequential requirements, we designed an injectable composite system comprising glycyrrhizic acid (GA), Zn^2+^, black phosphorus (BP), silk fibroin methacrylate (SilMA), and osteogenically induced bone microtissues (BO). The conceptual advance lies in combining a pre-induced, three-dimensional osteogenic microtissue with a functional hydrogel that locally retains and protects the living unit while providing antibacterial and microenvironment-modulating cues. This design differs from acellular multifunctional hydrogels, which provide material-derived signals without a pre-organized living osteogenic unit, and from previously reported microtissue/organoid delivery systems developed mainly for noninfected defects. In the “soil-seed-nutrient” framework, the GA/SilMA matrix is designated the locally retained “soil,” the day-7 osteogenic BO is the living “seed,” and Zn^2+^/BP are the antibacterial and regenerative “nutrient cues.” Three-dimensional bone microtissues preserve cell–cell interactions and a tissue-like osteogenic phenotype, but require protection from bacterial invasion, inflammatory stress, and insufficient vascular support after implantation [[23], [24], [25]].

In this study, we evaluated whether GA-Zn^2+^/BP/BO@SilMA could sequentially coordinate bacterial suppression, macrophage remodeling, vascular regeneration, and bone formation. The system was characterized physicochemically and assessed using in vitro antibacterial, macrophage, angiogenic, and osteogenic assays, a mouse subcutaneous infection model, and an infected rat caudal intervertebral defect model. We hypothesized that temporally coordinated antibacterial activity, inflammatory resolution, and angiogenesis–osteogenesis coupling would create a regenerative microenvironment capable of enhancing repair of infected intervertebral defects.

## 2 Materials and methods

### 2.1 Materials

The materials and reagents used in this study are as follows: glycyrrhizic acid micelles (GA, Xianfeng Nano, China), black phosphorus quantum dots (BP, Xianfeng Nano, China), zinc chloride (Macklin, China), LAP photoinitiator (Aladdin, China), PBS buffer (Servicebio, China), 4% paraformaldehyde (Servicebio, China), ECL(Servicebio, China)2.5% glutaraldehyde (Solarbio, China), fetal bovine serum (FBS, Merck, Australia), penicillin-streptomycin solution (Beyotime, China), 0.25% trypsin-EDTA (Gibco, USA), CCK-8 assay kit (Beyotime, China), DAPI stain (Beyotime, China), anti-fluorescence quenching mounting medium (Beyotime, China), bacterial culture medium (Beyotime, China), and LB agar plates (Beyotime, China).

### 2.2 Preparation of SilMA and GA-Zn^2+^/BP@SilMA hydrogels

Silk fibroin was isolated from natural cocoons by degumming in Na_2_CO_3_ solution, followed by washing, drying, and dissolution in 9.3 M LiBr at 60 °C. The resulting silk fibroin solution was dialyzed against deionized water for 4 days, filtered, frozen, and lyophilized. SilMA was prepared by adding glycidyl methacrylate at a ratio of 1 mL per 4 g of silk fibroin and reacting at 60 °C for 3 h under continuous stirring. The reaction product was dialyzed against deionized water for 4 days, filtered, frozen, and lyophilized. GA-Zn^2+^ coordination assembly and incorporation of BP, LAP, and BOs were performed subsequently.

To prepare 5 mL of hydrogel precursor, 1 mL of 2% (w/v) GA solution was mixed with 1 mL of 1% (w/v) ZnCl_2_ solution to form the GA-Zn^2+^ assembly. Subsequently, 100 μL of BP quantum-dot solution (0.05 mg/mL), 2.5 mL of 20% (w/v) SilMA, 250 μL of 1% (w/v) LAP, and 150 μL of deionized water were added. The final hydrogel composition consisted of 0.40% (w/v) GA, 0.20% (w/v) ZnCl_2_, 1.0 μg/mL BP, 10% (w/v) SilMA, and 0.05% (w/v) LAP. The precursor was photo-crosslinked for approximately 3 min using 390 nm violet light.

### 2.3 Preparation of bone microtissues and hydrogel-integrated constructs

Rat bone marrow mesenchymal stem cells (rBMSCs, Procell Life, No.CP-R131) were used to generate bone microtissues through centrifugal self-assembly followed by osteogenic induction. Briefly, rBMSCs were digested, collected, and centrifuged at 1000 rpm for 5 min. The cell pellet was resuspended in complete medium, and 100 μL of cell suspension containing approximately 2 × 10^4^ cells was added into each well of a 384-well plate. The plate was sealed, centrifuged at 1000 rpm for 5 min, and incubated at 37 °C for 8 h to allow cell aggregation.

Agarose-coated culture dishes were prepared by dissolving 100 mg agarose in 20 mL deionized water to reduce adhesion during microtissue culture, followed by heating, solidification, and ultraviolet sterilization. The formed rBMSC aggregates were transferred onto agarose-coated dishes and cultured in osteogenic induction medium, which was changed every 2 days. After 7 days of induction, compact bone microtissues with an average diameter of approximately 400 μm were obtained.

Bone microtissues were incorporated into the hydrogel precursor at 50 BOs/mL. Because each BO contained approximately 2 × 10^4^ rBMSCs, this loading corresponded to approximately 1 × 10^6^ cells/mL. Each 100-μL animal implant therefore contained 5 BOs (approximately 1 × 10^5^ cells). The preparation sequence was SilMA synthesis and purification, separate GA-Zn^2+^ coordination assembly, addition of BP/SilMA/LAP, loading of BOs, and 390 nm photocrosslinking. The final construct was consistently designated GA-Zn^2+^/BP/BO@SilMA.

### 2.4 Hydrogel characterization

Hydrogels were freeze-dried, fractured to expose the internal cross-section, sputter-coated with gold, and imaged by field-emission scanning electron microscopy (SEM, Sigma 360, ZEISS, Germany). Energy-dispersive X-ray spectroscopy (EDS, Sigma 360, ZEISS, Germany) mapping was used to examine the distribution of C, O, Zn, and P.Fourier transform infrared spectroscopy(FTIR, Nicolet iS50, Thermo Fisher Scientific, USA) was performed with 32 scans in the range of 4000–400 cm^−1^ at a resolution of 4 cm^−1^; X-ray diffraction patterns (XRD, SmartLab SE, Rigaku Corporation, Japan) were recorded from 5° to 90°. Surface morphology, roughness parameters (Ra and Rq), and local nanoscale Young's modulus were characterized using atomic force microscopy(AFM, Dimension Icon, Bruker, Germany).

To analyze Zn^2+^ release, 1 mL of hydrogel was immersed in 15 mL of DMEM at 37 °C. At each predetermined time point, 10 mL of medium was removed and replaced with fresh DMEM, leaving 5 mL in the container. Zn^2+^concentrations were measured by ICP-OES (5800 ICP-OES, Agilent Technologies, USA) on days 1, 3, 7, and 11. To analyze hydrogel degradation, 1 mL of hydrogel was placed in a 24-well plate and treated with 2 mL of PBS or enzyme-containing PBS solution. The liquid was removed daily, the samples were dried and weighed, and the degradation solution was replenished. The residual mass percentage was calculated as (W_t_/W_0_) × 100%.

### 2.5 Cytocompatibility evaluation

#### 2.5.1 Cell metabolic activity

rBMSCs were cultured in low-glucose DMEM supplemented with 10% FBS and 1% penicillin-streptomycin at 37 °C in 5% CO_2_. For CCK-8 analysis, hydrogels were placed in Transwell inserts and rBMSCs were seeded in the lower chamber; thus, the 4, 8, and 12h measurements represent cell metabolic activity under indirect hydrogel exposure and not direct adhesion to the hydrogel. Additional CCK-8 measurements of cellular metabolic activity were performed at 24, 48, and 96 h, and the absorbance at 450 nm was recorded using an Epoch 2 Microplate Spectrophotometer (BioTek Instruments, USA).

#### 2.5.2 Cell morphology and spreading

To evaluate the cytoskeleton, rBMSCs in the lower chamber after 3 days of co-culture were fixed with 4% paraformaldehyde for 20 min, permeabilized with 0.1% Triton X-100 for 10 min, and washed three times with PBS. Subsequently, F-actin was stained with rhodamine-labeled phalloidin for 30 min, and cell nuclei were stained with DAPI for 15 min. Cell morphology and cytoskeletal structures were then observed using a confocal laser scanning microscope(CLSM, FLUOVIEW FV3000, Olympus Corporation, Japan).

### 2.6 In vitro antibacterial and antibiofilm assays

*Staphylococcus aureus* (Mingzhou Biotechnology, Cat. No. B81854) and *Escherichia coli* (provided by Renji Hospital, Shanghai Jiao Tong University School of Medicine) were cultured overnight in LB medium at 37 °C with shaking and diluted to the logarithmic growth phase before use.

#### 2.6.1 Bacterial morphology

Sterilized hydrogels were incubated with bacterial suspensions (1 × 10^6^ CFU/mL) at 37 °C for 12 h. After incubation, samples were gently rinsed, fixed in 2.5% glutaraldehyde at 4 °C, dehydrated through a graded ethanol series, dried, and examined by SEM.

#### 2.6.2 Colony-counting assay

After 12 h of incubation, planktonic bacteria and bacteria recovered from the hydrogel surface were collected separately, serially diluted, plated on LB agar, and incubated at 37 °C for 24 h. Bacterial burden was expressed as log_10_ (CFU/mL).

#### 2.6.3 Crystal-violet biofilm assay

To evaluate the ability of hydrogels to prevent biofilm formation, the hydrogels were incubated overnight with log-phase bacterial suspensions for 12 h. After incubation, the bacteria-exposed hydrogels were washed three times with sterile PBS, fixed for 30 min, and then stained with 0.1% (w/v) crystal violet for 15 min. Excess dye was removed, and images of the stained biofilms were captured. Subsequently, bound crystal violet was dissolved in 30% (v/v) acetic acid for 15 min, and absorbance was measured at 550 nm using a microplate reader. The experiment was performed in three independent biological replicates (n = 3).

#### 2.6.4 Live/dead bacterial staining

Hydrogel-treated bacteria were stained with SYTO 9 and propidium iodide according to the manufacturer's instructions and imaged to distinguish viable and membrane-compromised bacteria.

### 2.7 Evaluation of GA-Zn^2+^/BP@SilMA on macrophage phenotype regulation

#### 2.7.1 In vitro macrophage polarization

Bone marrow cells were collected from the femoral marrow cavities of two euthanized mice, mixed, and cultured for 7 days in medium supplemented with recombinant mouse M-CSF (50 ng/mL) to generate BMDMs. LPS (100 ng/mL) was added during the first 24 h of the hydrogel experiment, and the medium was subsequently replaced according to the experimental protocol without additional LPS. IL-4 (40 ng/mL, 24 h) was used as the induction regimen for M2-like macrophages. Independent cultures were collected on day 3 and day 7, respectively.

For immunofluorescence analysis, BMDMs were fixed, permeabilized, and blocked after 3 or 7 days of co-culture. Cells were incubated with antibodies against iNOS (ABclonal, A3774,1:200) and CD206 (ABclonal, A21014, 1:200), followed by fluorescent secondary antibodies and DAPI. Images were acquired by confocal microscopy and quantified using a prespecified analysis protocol.

Western blotting: BMDMs were lysed with RIPA lysis buffer containing protease inhibitors. Proteins were separated using an electrophoresis system and transferred onto a PVDF membrane via a wet transfer apparatus (Servicebio, China). After blocking, the membranes were incubated overnight at 4 °C with primary antibodies against iNOS, CD86, Arg-1, CD206, and GAPDH. All primary antibodies were purchased from ABclonal, used at the following dilutions: anti-iNOS (ABclonal, A3774, 1:1000), anti-CD86 (ABclonal, A1199, 1:500), anti-Arg-1 (ABclonal, A4923, 1:1000), anti-CD206 (ABclonal, A21014, 1:5000), and anti-GAPDH (ABclonal, AC001, 1:10,000). The membranes were washed three times with TBST, followed by incubation for 2 h with HRP-conjugated goat anti-rabbit IgG secondary antibody (ABclonal, AS014, 1:10,000). Protein bands were visualized and quantified using ImageJ software, with GAPDH as the internal control.

#### 2.7.2 Subcutaneous implant-associated infection model

We established a subcutaneous implant-associated infection model in mice to evaluate the in vivo antibacterial and immunomodulatory properties of hydrogel matrices. All animal procedures were approved by the Animal Ethics Committee of the Laboratory Animal Center, Shanghai Jiao Tong University (approval no. A2025343-001). Briefly, after anesthetizing the mice, their back hair was shaved and disinfected. Under sterile conditions, a subcutaneous pouch was created, into which sterilized hydrogel was injected. The hydrogel was then illuminated to induce in situ crosslinking. Subsequently, 10 μL of a *Staphylococcus aureus* suspension (1 × 10^8^ CFU/mL) was injected around the implanted hydrogel to establish a localized infection. Implants and surrounding tissues were collected on day 3 and day 7 for bacterial colony quantification and histological analysis.

Explanted hydrogels and surrounding tissues were homogenized in sterile PBS. Serial dilutions were plated on LB agar, incubated, and used for CFU quantification. Live/dead bacterial staining was also performed.

For histological analysis, skin tissues were fixed in 4% paraformaldehyde, dehydrated, embedded in paraffin, and sectioned. Hematoxylin and eosin staining was used to evaluate inflammatory infiltration and fibrous tissue thickness. Immunofluorescence was performed to detect macrophage phenotypic markers using anti-iNOS(ABclonal, A3774, 1:500) and anti-CD206(ABclonal, A21014, 1:300) antibodies, followed by incubation with fluorescently labeled secondary antibodies and DAPI counterstaining. Fluorescence was semiquantitatively analyzed using ImageJ.

### 2.8 Angiogenic evaluation under inflammatory conditions

HUVECs (QuiCell, H162) were cultured under inflammatory conditions in the presence of the indicated hydrogels. Endothelial tube formation was evaluated, and expression of VEGFA, VEGFB, and PDGFA was quantified by qRT-PCR(QuantStudio 5, Thermo Fisher Scientific, USA) using GAPDH as the reference gene and the 2^−^ΔΔCt method. Primer sequences for qRT-qPCR are listed in Supplementary Table S1.

### 2.9 Osteogenic differentiation under inflammatory conditions

An inflammatory co-culture model was established using conditioned medium from LPS-stimulated BMDMs. rBMSCs or bone microtissues were cultured with the indicated hydrogel formulations in a Transwell system, and the conditioned medium was replaced every 2 days.

Early osteogenic differentiation was evaluated by alkaline-phosphatase staining and activity assays after 7 days. Matrix mineralization was evaluated by Alizarin Red S (Beyotime, China) staining after 14 days, followed by dye extraction and spectrophotometric quantification at 562 nm.

Osteogenic protein expression in bone microtissues was assessed by immunofluorescence staining for OCN (ABclonal, A6205, 1:200) and OPN (ABclonal, A23658, 1:200) and by western blotting for RUNX2(Abcam, ab192256,1:1000), OCN (ABclonal, A6205, 1:1000), and GAPDH (ABclonal, AC001, 1:10,000).

### 2.10 Infected rat caudal intervertebral defect model

Sixty male Sprague-Dawley rats (approximately 250 g) were randomly divided into five groups (12 rats per group): control group, SilMA group, GA-Zn^2+^@SilMA group, GA-Zn^2+^/BP@SilMA group, and GA-Zn^2+^/BP/BO@SilMA group. Before surgery, six animals from each group were assigned to the 8-week observation group, and the other six to the 12-week observation group. A defect measuring 4 × 4 mm was created at the intervertebral level between the second and third caudal vertebrae, followed by local injection of 10 μL of *Staphylococcus aureus* (1 × 10^8^ CFU/mL). Approximately 10 min later, 100 μL of the designated material was filled into the defect site and in situ photo-crosslinked. A portion of the native intervertebral disc tissue was preserved, and no cage was used.

### 2.11 Micro-CT and histological evaluation

The collected caudal vertebrae specimens were fixed in 4% paraformaldehyde and scanned using a micro-CT system. Three-dimensional reconstructed images and sagittal plane images were generated to assess bone regeneration and intervertebral fusion. Regions covering the intervertebral defect area were selected for quantitative analysis, measuring bone volume/total volume ratio (BV/TV), bone mineral density (BMD), trabecular number (Tb. N), and residual intervertebral-space length.

After micro-CT scanning, samples were decalcified, dehydrated, embedded, and sectioned. Hematoxylin and eosin staining was used to assess tissue continuity and defect repair, and Masson's trichrome staining was used to evaluate collagen deposition and bone-matrix formation.

### 2.12 Transcriptomic analysis

RNA sequencing(Shanghai Majorbio Bio-pharm Technology) was performed on untreated BOs and BOs treated with GA-Zn^2+^/BP@SilMA, with three biological replicates per group. High-quality RNA (RQN > 6.5, OD_260_/_280_ = 1.8–2.2, OD_260_/_230_ ≥ 2.0, total RNA > 1 μg) was used to construct libraries using the Illumina Stranded mRNA Prep Ligation Kit, followed by paired-end 150 bp sequencing on the NovaSeq X Plus platform. A total of 42.99 Gb of clean data were obtained, averaging 7.17 Gb per sample, with a Q30 value exceeding 95.82%. Sequencing data were processed using fastp, HISAT2, StringTie, and RSEM. Differentially expressed genes (DEGs) were identified via DESeq2 analysis, with selection criteria set at |log_2_FC| ≥ 1 and FDR < 0.05. GO, KEGG, heatmaps, volcano plots, and GSEA analyses were conducted on the Majorbio cloud platform.

### 2.13 Statistical analysis

Data are presented as mean ± standard deviation (SD) and analyzed using GraphPad Prism 10. Normality and homogeneity of variance were assessed prior to parametric tests. Comparisons among multiple groups were performed using one-way analysis of variance (ANOVA), followed by Tukey's multiple comparisons test. The biological sample size (n) represents independent biological replicates or individual animals, as specified in the figure legends. Samples and animals were randomly assigned to experimental groups (n > 3). Analyses were based on complete available datasets, with missing data not imputed. A two-sided P value less than 0.05 was considered statistically significant.

## 3 Results and discussion

### 3.1 Physicochemical characterization of the anti-infective hydrogel

**Fig. 1:**
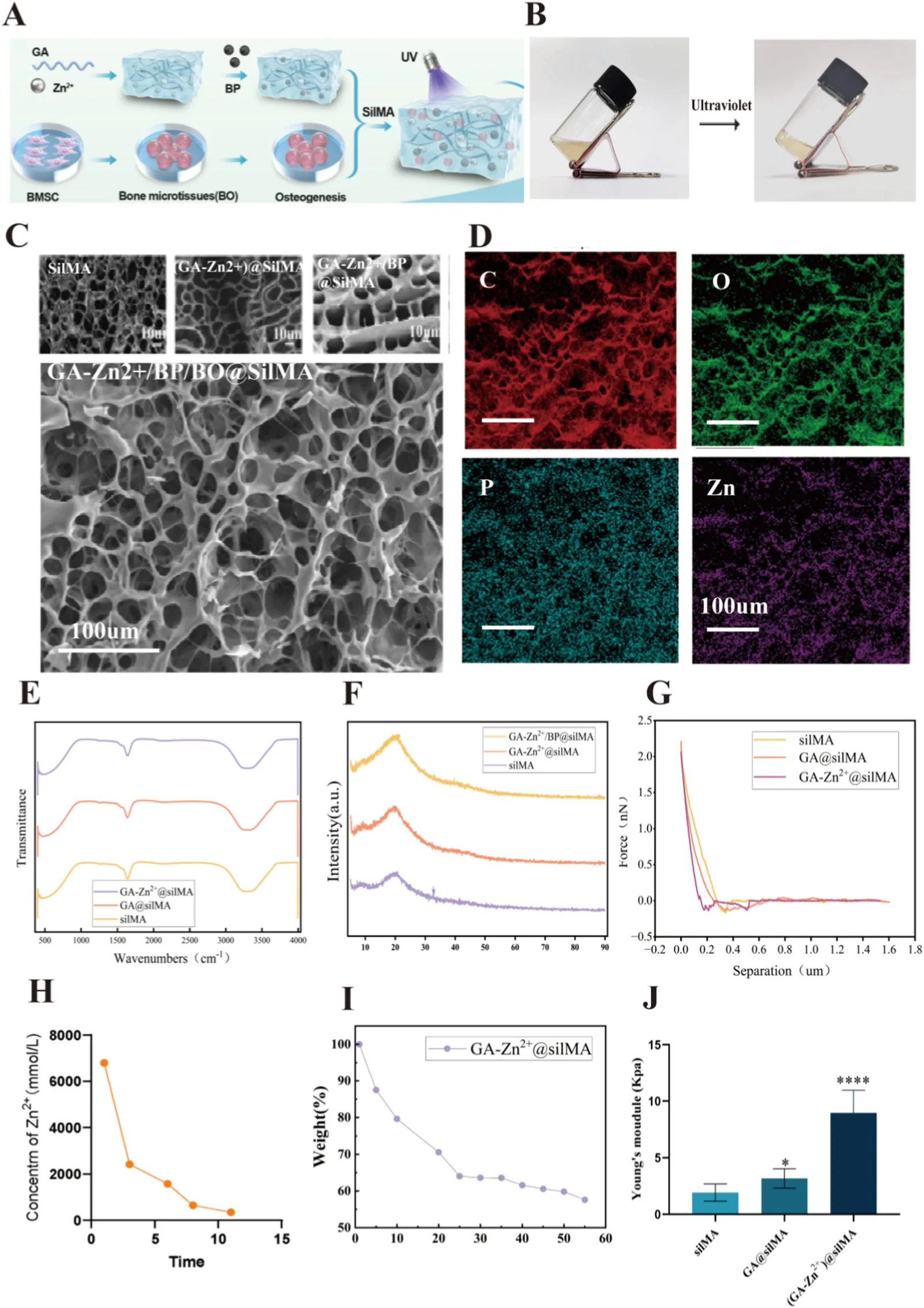
**Physicochemical and mechanical characterization of the hydrogel formulations.** **A)** Schematic illustration of the preparation of GA–Zn^2+^/BP@SilMA and incorporation of osteogenic bone microtissues (BO). **B)** Photographs of the hydrogel precursor before and after ultraviolet. **C)** Scanning electron microscopy (SEM) images of SilMA, GA–Zn^2+^@SilMA, GA–Zn^2+^/BP@SilMA, and GA–Zn^2+^/BP/BO@SilMA hydrogels. **D)** Energy-dispersive X-ray spectroscopy (EDS) elemental maps of C, O, P, and Zn in the composite hydrogel. **E)** Fourier-transform infrared spectroscopy (FTIR) spectra of the different hydrogel formulations. **F)** X-ray diffraction (XRD) patterns of the different hydrogel formulations. **G)** Atomic force microscopy (AFM) force–separation curves. **H)** Zn^2+^ release profile. **I)** Degradation behavior of the hydrogel. **J)** Young's modulus of the different hydrogel formulations. Data are presented as mean ± SD. *p < 0.05 and ****p < 0.0001.

Previous studies have revealed that divalent metal ions such as Zn^2+^ and Cu^2+^ can induce self-assembly of GA at low concentrations [26]. Schematic illustration of GA and Zn^2+^ undergoing the first stage of chemical cross-linking (Fig. 1A). The precursor formed a stable gel after photocrosslinking (Fig. 1B). SEM showed interconnected porous structures across all formulations, and EDS mapping confirmed the distribution of C, O, Zn, and P without obvious elemental aggregation (Fig. 1C, D and S1A). GA-Zn^2+^/BP@SilMA also adhered to glass, metal, and paper substrates (Fig. S2A).

FTIR spectra retained the characteristic amide bands of the SilMA matrix, while changes in the 3200-3500 and 1030-1150 cm^−1^ regions were consistent with GA incorporation and strengthened coordination/noncovalent interactions (Fig. 1E). XRD patterns remained broad and predominantly amorphous after incorporation of GA-Zn^2+^ and BP (Fig. 1F), indicating the absence of a dominant new crystalline phase.

AFM showed increased surface roughness and local nanomechanical stiffness after incorporation of GA-Zn^2+^ and BP(Fig. S1B and C). The measured surface modulus increased from approximately 1.5–2.0 kPa for SilMA to 3–4 kPa for GA@SilMA and 8–10 kPa for GA-Zn^2+^@SilMA (Fig. 1J). These findings support reinforcement of the local hydrogel network; however, they do not demonstrate that the hydrogel independently provides load-bearing intervertebral stability.

The Zn^2+^ concentration decreased from 6802.64 ± 86.75 μg/L on day 1 to 2488.56 ± 92.99 μg/L on day 3, 1421.66 ± 14.05 μg/L on day 7, and 342.43 ± 20.46 μg/L on day 11 (Fig. 1H), corresponding approximately to 104, 38, 22, and 5 μM, respectively. This curve represents the measured concentration in the medium over time. The early concentration was above commonly reported osteogenic ranges and may primarily support antibacterial activity, whereas the day 3–7 concentrations overlapped with reported immunoregulatory and osteogenic ranges [27,28]. Silk fibroin exhibits excellent biodegradability [29]. Fig. 1I shows the quantitative residual mass degradation from day 0 to day 55, while Fig. S2B provides a schematic illustration of the degradation process in PBS and Pronase E from day 0 to day 25.

### 3.2 Characterization of osteogenic bone microtissues

Osteogenic bone microtissues were generated by centrifugal aggregation followed by osteogenic induction (Fig. S3B). OCN immunofluorescence was stronger on day 7 than on day 5 (Fig. S3D). RUNX2, a key transcriptional regulator of osteoblast-lineage commitment, was also upregulated, consistent with continued progression of osteogenic differentiation [[30], [31], [32]]. OCN, RUNX2, OSX, and OPN expression increased from day 5 to day 7, indicating progression toward an osteogenic phenotype (Fig. S3C–I). Day-7 Bone microtissues were therefore selected for incorporation into the hydrogel.

### 3.3 Cytocompatibility of hydrogel matrix

**Fig. 2:**
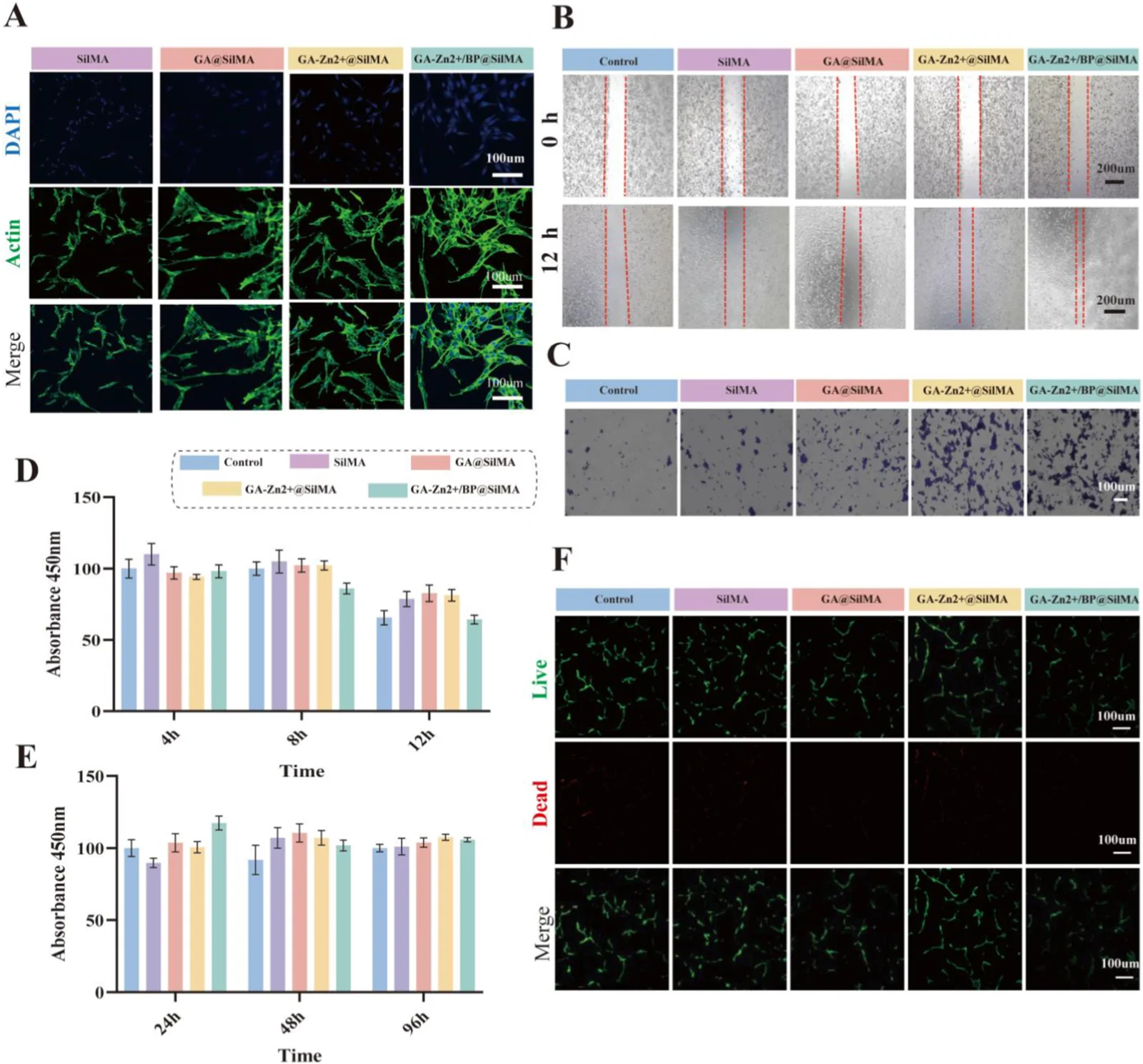
**Cell compatibility, migration ability, metabolic activity, and survival rate of rBMSCs co-cultured with hydrogels.** **A)** Representative fluorescence images of the rBMSC cytoskeleton after indirect exposure to the different hydrogel formulations. **B)** Representative scratch-wound images showing rBMSC migration at 0 and 12 h. **C)** Transwell migration assay. **D)** CCK-8-derived metabolic activity of rBMSCs at 4, 8, and 12 h. **E)** CCK-8-derived metabolic activity of rBMSCs at 24, 48, and 96 h. **F)** Live/dead staining of rBMSCs after co-culture with different hydrogels.

rBMSCs maintained ordered F-actin and nuclear morphology on all hydrogels. Cells co-cultured with GA-Zn^2+^/BP@SilMA exhibited significant spreading and elongated pseudopodia extension (Fig. 2A). Scratch and Transwell assays demonstrated enhanced cell migration ability in the functionalized hydrogel group (Fig. 2B and C). CCK-8 analysis showed no significant decline in cellular metabolic activity within 4 days, and live/dead staining revealed that cells were predominantly viable (Fig. 2D–F). These results support the excellent biocompatibility of GA-Zn^2+^/BP@SilMA.

### 3.4 Antibacterial and immunomodulatory activities of the hydrogel

The hydrogel matrix was designed to protect the osteogenic microtissues from bacterial and inflammatory stress while reshaping the local immune environment. Its antibacterial and immunomodulatory macrophage effects were therefore evaluated before analysis of angiogenic and osteogenic outcomes.

#### 3.4.1 In vitro antibacterial and antibiofilm activity

**Fig. 3:**
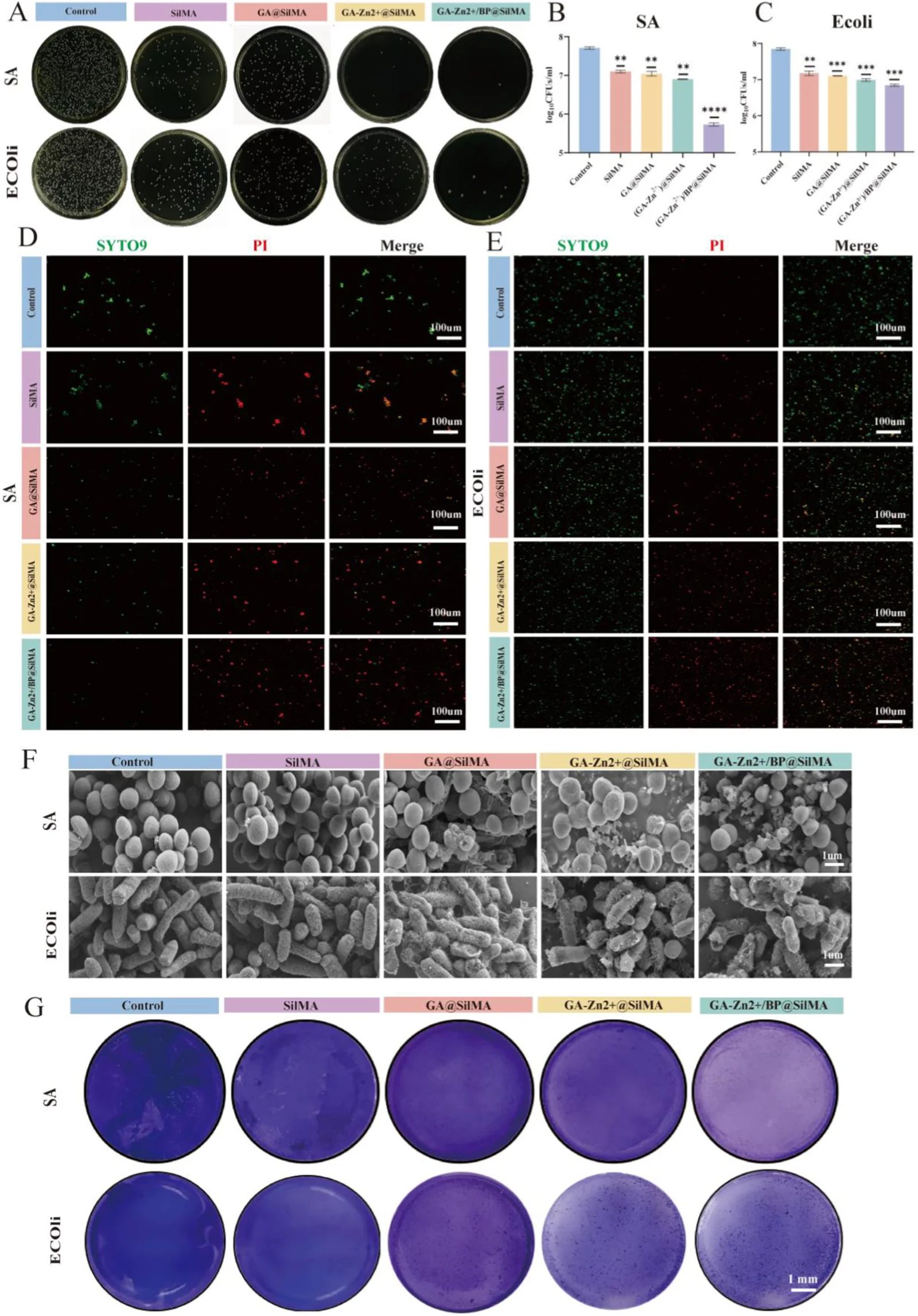
**In vitro antibacterial activity and biofilm inhibition.** **A)** Representative agar plates of *Staphylococcus aureus* and *Escherichia coli* after treatment with different hydrogels. **B, C)** Number of surviving *Staphylococcus aureus* and *Escherichia coli* expressed as log_10_ CFU/mL. **D, E)** Live/dead staining images of *Staphylococcus aureus* and *Escherichia coli* after various treatments. **F)** SEM images of *Staphylococcus aureus* and *Escherichia coli* after different treatments. **G)** Crystal violet staining after 12 h of hydrogel exposure. Data are presented as mean ± standard deviation. *p < 0.05, **p < 0.01, ***p < 0.001, ****p < 0.0001.

In the bacteria-hydrogel co-culture system, the GA–Zn^2+^/BP@SilMA group showed the most significant reduction in bacterial load. For *Staphylococcus aureus*, the bacterial count in the control group decreased from 7.71 ± 0.03 log_10_ CFU/mL to 5.73 ± 0.03 log_10_ CFU/mL in the GA–Zn^2+^/BP@SilMA group. For *Escherichia coli*, the corresponding bacterial counts were 7.85vb ± 0.03 and 6.84 ± 0.03 log_10_ CFU/mL, respectively (Fig. 3A–C). Zn and BP quantum dots exhibit direct antibacterial activity [33]. Live/dead staining showed an increased proportion of membrane-damaged bacteria, scanning electron microscopy revealed bacterial shrinkage and surface structural damage, while crystal violet staining indicated reduced surface-associated biomass in the groups containing Zn^2+^/BP (Fig. 3D–G). The reduction in *Staphylococcus aureus* load was greater than that of *Escherichia coli*, indicating that the composite hydrogel exhibits varying sensitivity to different bacterial species. Since these bacterial tests were conducted in the absence of macrophages, the reduction in bacterial load, survival rate, and surface-associated biomass directly demonstrates that the material itself possesses antibacterial activity independent of macrophage-mediated clearance.

#### 3.4.2 In vitro macrophage immune regulatory response

**Fig. 4:**
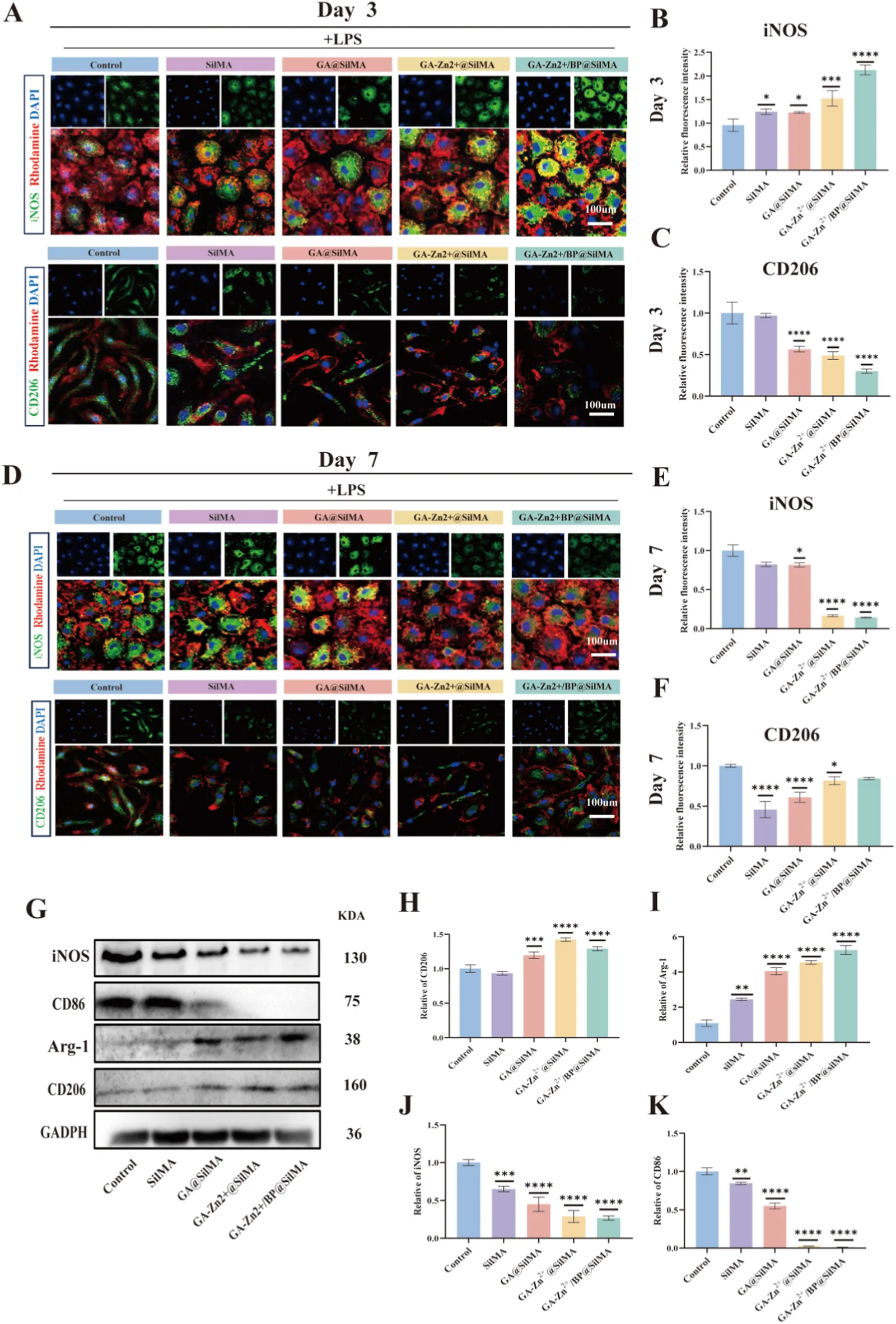
**Time-dependent macrophage-associated marker profiles after exposure to different hydrogels.** **A)** Representative immunofluorescence images of iNOS and CD206 in macrophages exposure to the different hydrogels. **B, C)** Quantification of iNOS and CD206 fluorescence intensity on day 3. **D)** Representative immunofluorescence images of iNOS and CD206 in macrophage exposure to the different hydrogels. **E, F)** Quantification of iNOS and CD206 fluorescence intensity on day 7. **G)** Western blot analysis of iNOS, CD86, Arg-1, and CD206. H–K) Quantification of CD206, Arg-1, iNOS, and CD86 protein expression, respectively.Data are presented as mean ± SD. *p < 0.05, **p < 0.01, ***p < 0.001, and ****p < 0.0001.

The polarization switch of macrophages from the pro-inflammatory M1 phenotype to the anti-inflammatory M2 phenotype can prevent the inflammation-induced reduction in osteogenesis rate [34]. To characterize the dynamic changes in macrophages, iNOS and CD86 were used as M1-like associated markers, while CD206 and Arg-1 served as M2-like associated markers. At day 3, the hydrogel group containing GA showed enhanced iNOS staining, while CD206 signaling remained relatively low, indicating that the early macrophage phenotype was predominantly characterized by M1-like markers (Fig. 4A–C). At day 7, IF and Western blot analyses showed lower expression levels of iNOS and CD86, along with elevated expression of CD206 and Arg-1 in the functionalized hydrogel group, with the GA-Zn^2+^/BP@SilMA group exhibiting the most prominent M2-like phenotype markers (Fig. 4D–K). These findings collectively indicate that the macrophage-associated marker profile exhibits a time-dependent remodeling pattern, characterized initially by dominance of M1-like signals, followed by a gradual shift toward M2-like signals. In a diabetic wound model, Lu et al. also demonstrated that a multifunctional thiamine-kaempferol hydrogel loaded with ameloflavone exhibits antibacterial and anti-inflammatory activity, thereby accelerating tissue repair [35]. Because LPS stimulation was limited to the initial 24 h, the attenuation of inflammation-related markers on day 7 may partly reflect an adaptation or resolution process occurring in cells after stimulus removal. However, all groups underwent identical stimulation and culture protocols, and the functionalized hydrogel group showed more pronounced changes in marker expression compared to the corresponding time-matched control group, supporting a material-associated immunomodulatory effect.

#### 3.4.3 In vivo antibacterial and immunomodulatory effects

**Fig. 5:**
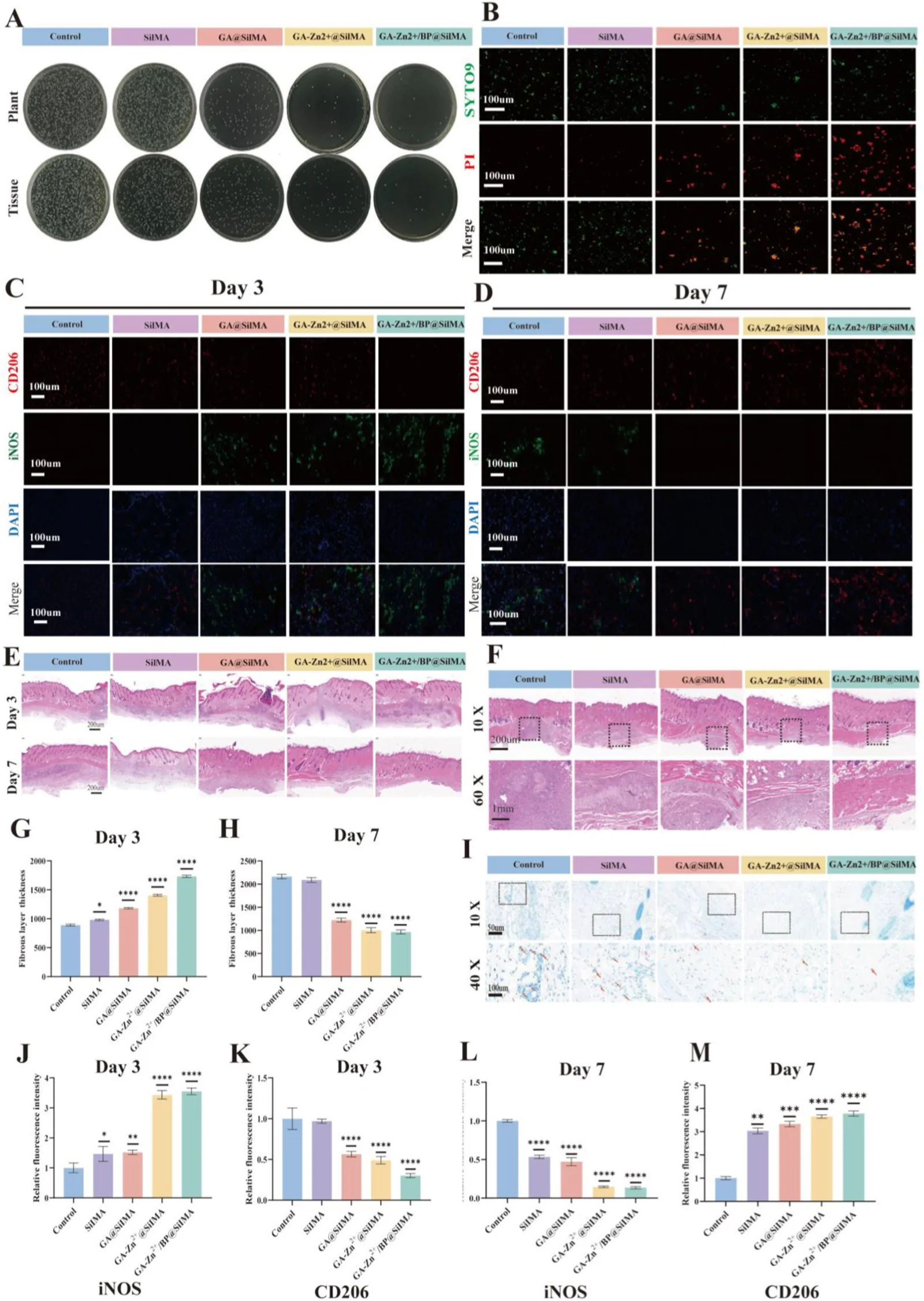
**In vivo bacterial load and peri-implant tissue response in a mouse subcutaneous implant infection model.** **A)** Representative agar plates showing bacteria recovered from the implants and surrounding tissues after the different treatments. **B)** Live/dead bacterial staining in peri-implant tissue; live and membrane-compromised bacteria were stained with SYTO9 and propidium iodide, respectively. **C, D)** Immunofluorescence staining of iNOS and CD206 in mouse subcutaneous tissue on days 3 and 7. **E, F)** H&E staining of peri-implant tissue on days 3 and 7. **G, H)** Quantification of peri-implant fibrous-tissue thickness on days 3 and 7. I) Giemsa staining of bacteria in peri-implant tissue. **J, K)** Quantification of iNOS and CD206 fluorescence intensity on day 3, respectively. **L, M)** Quantification of iNOS and CD206 fluorescence intensity on day 7, respectively. Independent animal cohorts were assessed at the two time points. Data are presented as mean ± SD. *p < 0.05, **p < 0.01, ***p < 0.001, and ****p < 0.0001.

In the subcutaneous infection mouse model, the recoverable *Staphylococcus aureus* load in the hydrogel group containing GA was lower than that in the control and SilMA groups, with the GA–Zn^2+^/BP@SilMA group showing the lowest load (Fig. 5A). Live/dead bacterial imaging and Giemsa staining showed a synchronized decrease in bacterial viability and residual bacterial signals, respectively (Fig. 5B,I). These bacteriological tests collectively support that the functionalized hydrogel group has a lower in vivo bacterial load. However, due to the simultaneous presence of implanted materials and an intact host immune response in the subcutaneous model, it is not possible to determine the individual contributions of the material's direct antimicrobial activity, inhibition of bacterial growth, and host immune clearance [36].

On day 3, immunofluorescence analysis of the sample groups showed higher iNOS signals and lower CD206 signals in the functionalized hydrogel group(Fig. 5C,J,K). In contrast, on day 7, another set of samples exhibited different marker characteristics: the functionalized hydrogel group displayed lower iNOS signals and higher CD206 signals, while the control group maintained strong iNOS signals (Fig. 5 D,L,M). Overall, the samples from day 3 and day 7 showed time-dependent differences in macrophage marker profiles, consistent with features of early inflammatory activation and subsequent attenuation of the inflammatory response [37].

H&E staining on day 3 showed acute inflammatory cell infiltration around the implant, along with the formation of a relatively thick fibroblast layer(Fig. 5E). In the early stages of implantation, the thicker layered structure observed in the GA-containing hydrogel group may reflect a prominent host response around the implant, including acute cellular recruitment and transient extracellular matrix deposition, rather than evidence of mature fibrotic encapsulation or completed tissue repair [38]. On day 7, compared to the control and SilMA groups at the same time point, the GA@SilMA, GA-Zn^2+^@SilMA, and GA-Zn^2+^/BP@SilMA groups showed reduced inflammatory infiltration and thinner fibrous tissue layers, whereas significant inflammatory infiltration and thicker peri-implant tissues were still observed in the control and SilMA groups(Fig. 5E). The fiber layer thickness in the GA–Zn^2+^/BP@SilMA group was 1731.87 ± 20.06 μm in the experimental animal group on day 3 and 966.33 ± 42.72 μm on day 7(Fig. 5G and H). These histological findings indicate that different experimental animal groups at day 3 and day 7 exhibited distinct characteristics of tissue response around the implants, with the hydrogel group showing a less persistent fibroinflammatory encapsulation reaction at the later time point(Fig. 5F). Overall, the subcutaneous model supports that this hydrogel possesses antibacterial and immunomodulatory activities. However, these experimental results do not fully align with the actual progression following a complete spinal infection, and further experimental validation is needed.

### 3.5 Immune–vascular–osteogenic regulation under inflammatory conditions

The vascular system plays a crucial role in bone tissue development, repair, and remodeling by regulating local nutrient supply, cell recruitment, and signaling, thereby helping to maintain the balance between osteogenesis and osteoclastogenesis [[39], [40], [41]]. GA-Zn^2+^@SilMA and GA-Zn^2+^/BP@SilMA promoted more extensive HUVEC network formation than the control, SilMA, and GA@SilMA groups after 12 h (Fig. S5A and E). VEGFA, VEGFB, and PDGFA expression was also increased in the functionalized hydrogel groups (Figs. S5B, C, D). The GA@SilMA group showed a smaller pro-angiogenic effect, suggesting that immune modulation may contribute in addition to direct Zn^2+^/BP signaling. These data support pro-angiogenic activity but do not yet establish the molecular pathway.

**Fig. 6:**
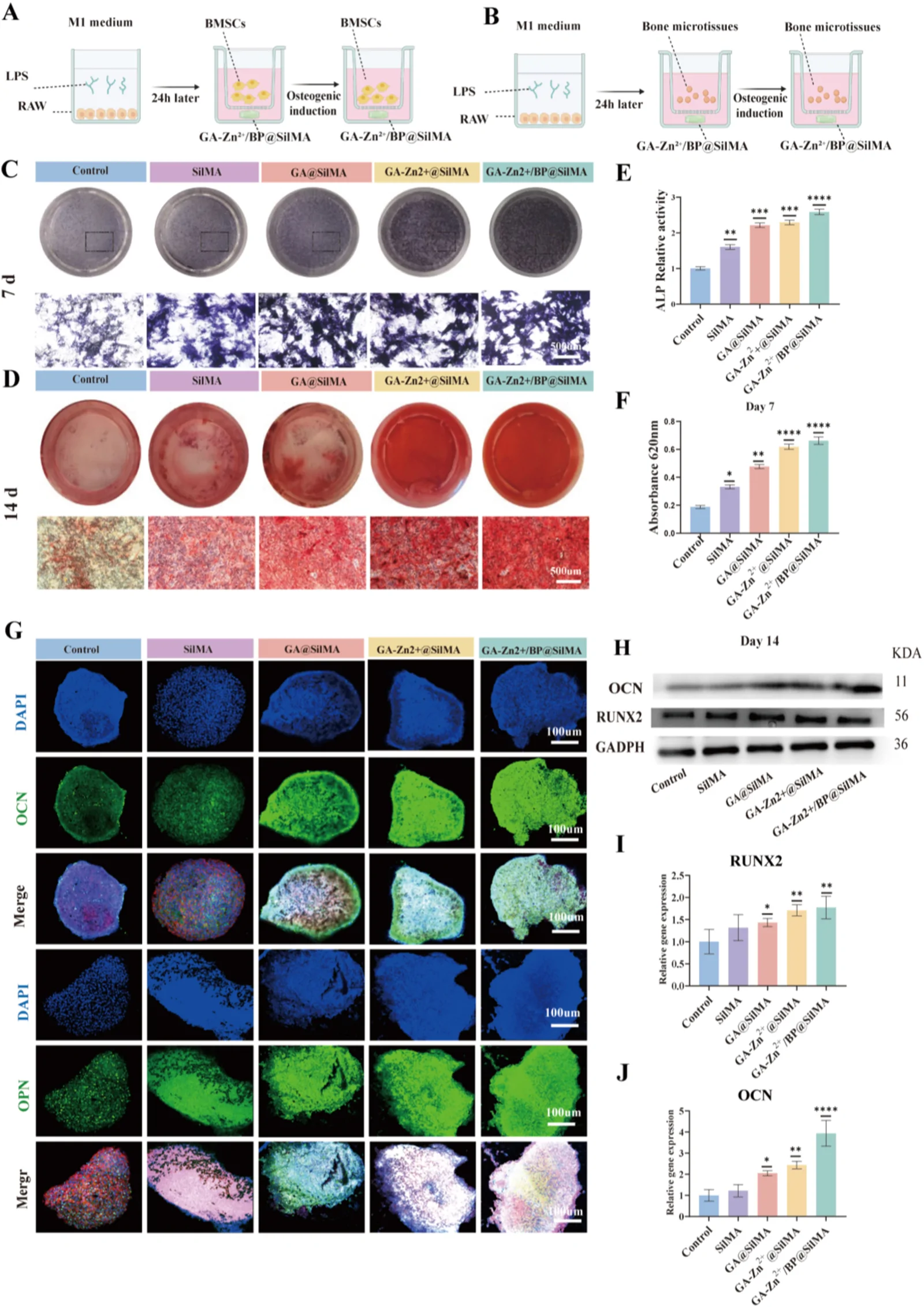
**Osteogenic response supported by hydrogel under conditional inflammatory environment of RAW264.7 macrophages.** **A, B)** Schematic illustrations of the inflammatory culture systems established using conditioned medium from LPS-stimulated RAW264.7 macrophages to evaluate osteogenic differentiation of rBMSCs **(A)** and osteogenic bone microtissues (BO) **(B)**. **C, E)** Representative ALP)staining and quantitative analysis of ALP activity on day 7. **D, F)** Representative ARS staining and quantitative analysis of mineral deposition on day 14. **G)** Immunofluorescence staining of OCN and OPN in BO after the different treatments. **H)** Western blot of RUNX2 and OCN expression. I,J) Quantification of RUNX2 and OCN. Data are presented as mean ± SD. *p < 0.05, **p < 0.01, ***p < 0.001, and ****p < 0.0001.

The inflammatory co-culture model was employed to evaluate the immunoregulatory effect on bone formation (Fig. 6A and B).In the inflammatory co-culture model, hydrogel treatment increased ALP activity at day 7 and mineral deposition at day 14, with the strongest responses in the GA-Zn^2+^/BP@SilMA groups cultured with BMDMs (Fig. 6C, D, E, F). The findings indicate that the composite hydrogel preserves osteogenic differentiation under inflammatory conditions. A macrophage-dependent mechanism remains plausible but requires conditioned-medium transfer, macrophage depletion, or pathway-blocking experiments.

Bone microtissues cultured with functionalized hydrogels showed increased OCN and OPN immunofluorescence and higher RUNX2 and OCN protein expression, particularly in GA-Zn^2+^/BP@SilMA (Fig. 6G,H,I,J and S5 F,G) [42]. Thus, the hydrogel supported the osteogenic phenotype of the encapsulated microtissues under inflammatory conditions.

Overall, endothelial and osteogenic experiments support a coordinated role of angiogenesis and osteogenic responses. Current studies indicate parallel and potentially interactive effects, but a complete macrophage-mediated immune-angiogenesis-osteogenesis cascade has not yet been confirmed [43].

### 3.6 Bone regeneration and intervertebral bridging in vivo

**Fig. 7:**
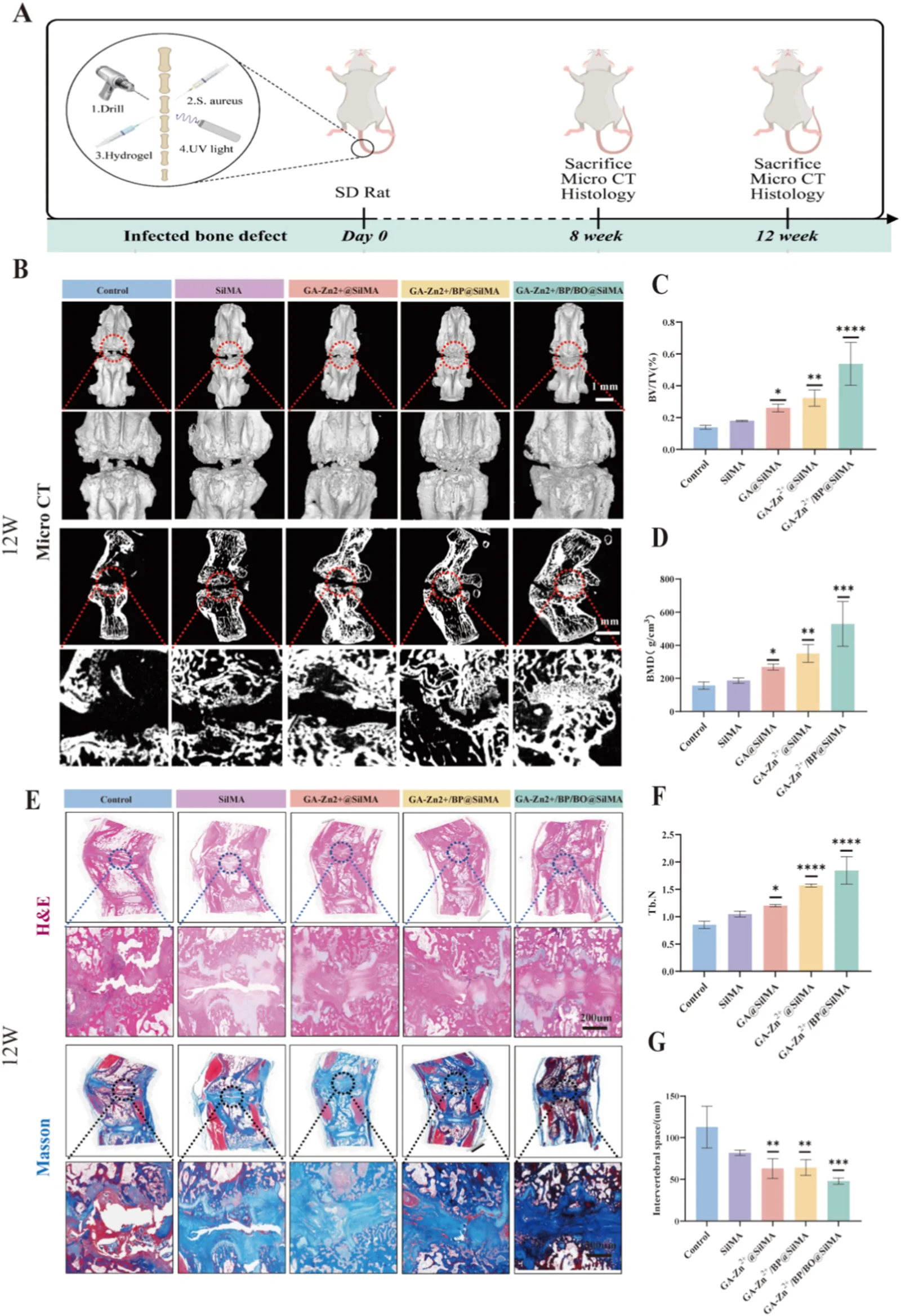
**Structural bony bridge formation in a rat Co2/Co3 intervertebral defect model with bacterial contamination.** **A)** Schematic illustration of the model establishment and evaluation schedule at weeks 8 and 12. **B)** Representative three-dimensional micro-CT reconstructions and sagittal images of the defect area at week 12. **C, D, F)** Quantitative micro-CT analysis results for bone volume/total volume (BV/TV), bone mineral density (BMD), and trabecular number (Tb.N) at week 12. E) H&E staining and Masson staining of the defect area at week 12. **G)** Quantitative analysis of residual intervertebral disc space at week 12. Data are presented as mean ± standard deviation (n = 6 animals per group). *p < 0.05, **p < 0.01, ***p < 0.001, ****p < 0.0001.

A bacteria-contaminated rat Co2/Co3 caudal intervertebral defect model was used to evaluate bone regeneration and structural bridging (Fig. 7A). At week 8, micro-CT revealed obvious intervertebral structural bridging only in the GA-Zn^2+^/BP/BO@SilMA group (Fig. S7A). Consistent with this observation, this group exhibited higher bone volume fraction (BV/TV) and bone mineral density (BMD) compared to the other groups (Fig. S7B and C). At 12 weeks, GA-Zn^2+^/BP/BO@SilMA showed the most extensive defect bridging and new bone formation on micro-CT (Fig. 7B). BV/TV, BMD, and Tb.N were higher than those in the other groups (Fig. 7C, D, and F), while H&E and Masson staining demonstrated greater tissue continuity, collagen deposition, and bone-matrix formation (Fig. 7E).Morphometric analysis revealed that, compared to the control group, the distance between adjacent vertebrae was reduced in the functionalized hydrogel groups, with the greatest reduction observed in the GA-Zn^2+^/BP/BO@SilMA group (Fig. 7G). Immunohistochemical results further showed that the group containing BO had higher positive areas for OCN and CD31, supporting stronger expression of osteogenic markers and formation of vasculature-related tissues at 12 weeks(Figure S6A, B,and C). Compared with GA-Zn^2+^/BP@SilMA, the incorporation of BO was associated with enhanced bone bridging, mineralization, osteogenesis, and vascularization. However, because dispersed rBMSCs and non-osteogenically induced spheroids were not included, the respective contributions of cell delivery, osteogenic pre-induction, and three-dimensional microtissue architecture could not be distinguished. Therefore, the present results support an overall regenerative contribution of BO but do not establish an architecture-specific effect. The BO used here represents an osteogenic microtissue rather than a vascularized or marrow-containing organoid. Future studies could incorporate endothelial or hematopoietic niche components to strengthen bone–vascular coupling and enable investigation of bone–marrow–immune interactions [44]. More broadly, bone and cartilage organoids and organ-on-chip platforms have advanced osteoarthritis modeling, therapeutic screening, and the development of cartilage-regenerative strategies, highlighting the broader translational potential of engineered musculoskeletal microtissues [42,45,46]. Since some of the native intervertebral discs were preserved, no fusion cages or structural bone grafts were implanted, and mechanical stability was not assessed, the observed endpoint should be described as radiographic and histological structural bridging, rather than clinically equivalent instrument-assisted intervertebral fusion. Since the hydrogel was implanted approximately 10 min after bacterial inoculation, this model represents intervention during acute bacterial contamination or early infection, rather than treatment for an established intervertebral disc space infection. Histological examination of the heart, liver, spleen, lung, and kidney showed no overt inflammatory infiltration or necrosis (Fig. S4), suggesting no obvious systemic histopathological toxicity under the tested conditions.

### 3.7 Transcriptomic analysis of bone microtissues

**Fig. 8:**
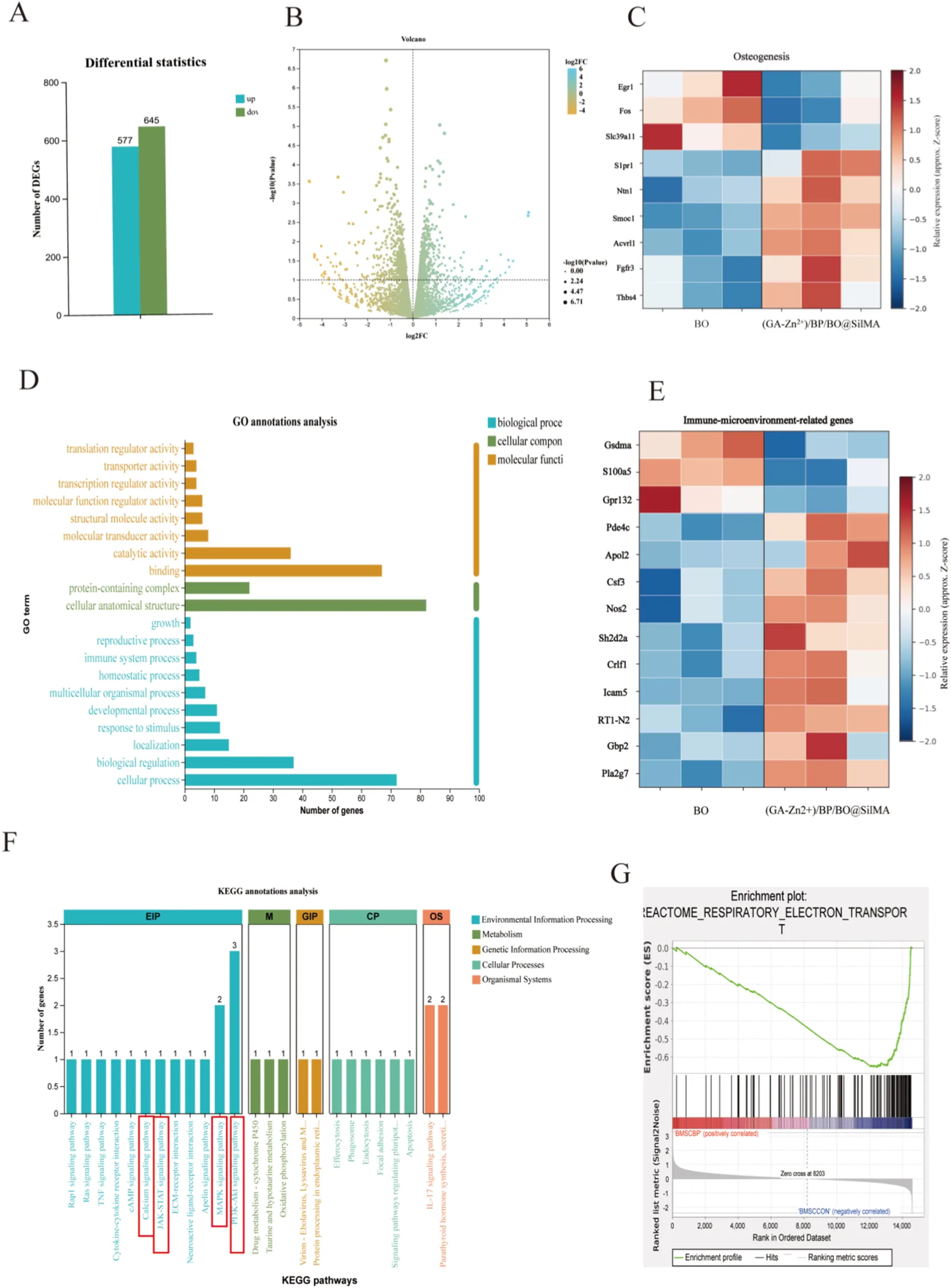
**Transcriptomic comparison of BO and GA–Zn^2+^/BP/BO@SilMA groups.** A) Number of differentially expressed genes (DEGs) upregulated and downregulated. **B)** Volcano plot showing the distribution of DEGs. **C)** Heatmap of genes related to osteogenesis. **D)** GO enrichment analysis of DEGs. **E)** Heatmap of genes associated with immune microenvironment. **F)** KEGG pathway enrichment analysis. **G)** Gene set enrichment analysis (GSEA) of the respiratory electron transport pathway. Each group consists of n = 3 biological replicates.

RNA sequencing identified 577 upregulated and 645 downregulated genes in hydrogel-treated bone microtissues relative to controls (Fig. 8A and B). The heatmap of the selected osteogenesis-related genes revealed significant differences in expression profiles between the BO group and the GA–Zn^2+^/BP/BO@SilMA group. S1pr1, Ntn1, Smoc1, Acvrl1, Fgfr3, and Thbs4 showed relatively higher expression in the composite hydrogel group, whereas Egr1, Fos, and Slc39a11 exhibited relatively lower expression (Fig. 8C). GO annotation categorized differentially expressed genes into three groups: biological processes, cellular components, and molecular functions. Among these, cellular processes and biological regulation were the main categories of biological processes, cell anatomical structure was the primary cellular component category, and binding and catalytic activity were the dominant molecular function categories (Fig. 8D).Enrichment analyses highlighted immune regulation, tissue remodeling, calcium signaling, PI3K–AKT, MAPK, JAK–STAT, cAMP signaling, and respiratory electron transport (Fig. 8E–G). These broad pathway signatures are consistent with changes in osteogenic differentiation, extracellular-matrix organization, and metabolic adaptation [[47], [48], [49]].

Transcriptomic data provide hypothesis-generating molecular associations rather than mechanistic proof. A single prioritized pathway should be validated by qRT-PCR and western blotting and tested functionally using a selective inhibitor or genetic perturbation. The sequencing dataset and complete analysis pipeline should also be deposited in a public repository.

## 4 Conclusion

GA–Zn^2+^/BP/BO@SilMA combines immunomodulatory hydrogels with osteogenic microtissues, not only leveraging the material's inherent antibacterial activity but also dynamically regulating macrophage phenotypic transition over time to promote angiogenesis and bone formation. In co-culture experiments with bacteria, this material effectively suppresses bacterial growth and prevents biofilm formation. At multiple independent evaluation time points, macrophage-associated markers consistently showed an initial M1-like phenotype, followed by a gradual shift toward an M2-like phenotype. We acknowledge that the macrophage markers assessed in this study may overlap, making it difficult to precisely define mutually exclusive activation states. Nevertheless, angiogenesis and osteogenic differentiation assays demonstrated that under inflammatory conditions, the material still supports endothelial network formation and osteoblastic differentiation. In an early-stage bacterial-contaminated murine Co2/Co3 intervertebral defect model, formulations containing Bone microtissues exhibited enhanced bone formation and structural bridging at 12 weeks, as confirmed by X-ray and histological evaluations. Transcriptomic analysis revealed potential associations with immune remodeling, extracellular matrix interactions, metabolic adaptation, and osteogenic signaling pathways, providing directions for future experimental validation. Additionally, this study has certain limitations: we did not confirm macrophage-mediated bacterial clearance, nor evaluated its efficacy against established mature biofilms or existing disc infections, and mechanical validation of vertebral fusion was not performed. Therefore, this system can be considered a potential local adjunctive strategy for early intervention and regenerative repair in bacterially contaminated intervertebral defects, but cannot replace actual clinical spinal fusion surgery.

## CRediT authorship contribution statement

**Shaokang Huang:** Writing – original draft, Visualization, Supervision, Software, Resources, Methodology, Investigation, Data curation. **Linli Li:** Writing – review & editing, Visualization, Methodology, Conceptualization. **Yichang Xu:** Writing – review & editing, Supervision, Software, Methodology, Investigation. **Jianming Huang:** Visualization, Software, Resources. **Yi Zhang:** Visualization, Software. **Shuying Chen:** Writing – review & editing, Supervision, Investigation, Data curation, Conceptualization. **Yu Chen:** Writing – review & editing, Funding acquisition, Data curation, Conceptualization. **Yushu Bai:** Writing – review & editing, Supervision, Funding acquisition, Data curation, Conceptualization. **Jie Shao:** Writing – review & editing, Investigation, Funding acquisition, Conceptualization.

## Declaration of generative AI

During the preparation of this work, the author(s) used Chatgpt for text revision. The author(s) reviewed and edited the output as needed and take full responsibility for the content of the published article.

## Declaration of competing interest

The authors declare that they have no known competing financial interests or personal relationships that could have appeared to influence the work reported in this paper.

## Data Availability

Data will be made available on request.
