# Supplementary material for: Hydrogel-integrated osteogenic microtissues promote repair of infected intervertebral defects through sequential immunomodulation
Source: Mater Today Bio. 2026 Sep 7;40:103657. doi: 10.1016/j.mtbio.2026.103657 (PMC13597281; doi:10.1016/j.mtbio.2026.103657)
Supplement: Supplementary file 1 [file mmc1.docx]

**Figure S1. Elemental composition and surface roughness of hydrogel formulations.**

**A)** Energy-dispersive X-ray spectroscopy (EDS) maps of SilMA, GA–Zn²⁺@SilMA, GA–Zn²⁺/BP@SilMA, and GA–Zn²⁺/BP/BO@SilMA. **B)** Three-dimensional atomic force microscopy (AFM) surface topography images of the four hydrogel formulations. **C)** Quantitative analysis of surface roughness parameters Ra and Rq. EDS confirms the detected elemental composition but does not itself prove uniform dispersion at the nanoscale. Data are presented as mean ± standard deviation. **p < 0.01, ****p < 0.0001.

**Figure S2. Adhesion and in vitro degradation of the functionalized hydrogel.**

**A)** Representative photographs showing adhesion of GA–Zn²⁺/BP@SilMA to metal, glass, and paper substrates. **B)** Representative photographs of hydrogel degradation in PBS and Pronase E-containing PBS from day 0 to day 25.

**Figure S3. Formation and osteogenic characteristics analysis of osteogenic bone microtissues (BO) derived from rBMSCs.**

**A)** Representative bright-field image of BO. Scale bar: 10 μm. **B)** Schematic illustration of BO formation and osteogenic induction. **C, D)** Immunofluorescence staining of OCN on days 5 and 7, respectively. **E)** Western blot analysis of OPN, OSX, RUNX2, and OCN after 5 and 7 days of osteogenic induction. **F–I)** Densitometric quantification of OPN, OCN, RUNX2, and OSX protein expression, respectively, normalized to GAPDH. Data are presented as mean ± SD.

**Figure S4. Systemic histopathological assessment after implantation in rats.** Representative H&E-stained sections of the heart, liver, spleen, lung, and kidney after the different treatments. Scale bar: 100 μm.

**Figure S5. Pro-angiogenic activity of functionalized hydrogels and quantitative analysis of osteogenic-marker fluorescence in BO.**

**A)** Representative images of HUVEC network formation after 12 hours of treatment with different hydrogel formulations. Scale bar: 20 μm. **B–D)** qRT-PCR analysis of VEGFA, VEGFB, and PDGFA mRNA expression. **E)** Quantitative analysis of HUVEC branch point number. **F, G)** Quantitative analysis of OCN and OPN fluorescence intensity in BO. Data are presented as mean ± standard deviation. *p < 0.05, **p < 0.01, ***p < 0.001, ****p < 0.0001.

**Figure S6. Immunohistochemical assessment of osteogenesis and vascularization in the rat caudal intervertebral defect model at 12 weeks.**

**A)** Representative immunohistochemical staining of OCN and CD31 in the defect areas after different treatments. Scale bar: 100 μm. **B, C)** Quantitative analysis of OCN-positive and CD31-positive areas. Data are expressed as mean ± standard deviation (n = 6 animals per group). *p < 0.05, ***p < 0.001, and ****p < 0.0001.

**Figure S7. Micro-CT assessment of the bacteria-contaminated rat Co2/Co3 caudal intervertebral defect model at 8 weeks.**

**A)** Representative 3D micro-CT reconstructions and sagittal images of the defect area at week 8. **B, C)** Quantitative analysis of bone volume/tissue volume (BV/TV) and bone mineral density (BMD). Data are presented as mean ± standard deviation (n = 6 animals per group). ***p < 0.001, ****p < 0.0001.

Supplementary Table S1

| Target gene | Direction | Primer sequence (5′ to 3′) |
| --- | --- | --- |
| β-actin | Forward | GCCTCGCCTTTGCCGAT |
| β-actin | Reverse | AGGTAGTCAGTCAGGTCCCG |
| VEGF-A | Forward | ACTGCCATCCAATCGAGACC |
| VEGF-A | Reverse | GCTCCAGGGCATTAGACAGC |
| VEGF-B | Forward | TCTCCCTGAGGCCATCATCA |
| VEGF-B | Reverse | TAGTGAGGGGAGGAAGAGCC |
| PDGF-A | Forward | CAGCGACTCCTGGAGATAGAC |
| PDGF-A | Reverse | GGAGGAGAACAAAGACCGCA |
